# Preparation and Characterization of Cyclotrimethylenetrinitramine (RDX) with Reduced Sensitivity

**DOI:** 10.3390/ma10080974

**Published:** 2017-08-21

**Authors:** Yuqiao Wang, Xin Li, Shusen Chen, Xiao Ma, Ziyang Yu, Shaohua Jin, Lijie Li, Yu Chen

**Affiliations:** 1 School of Materials Science and Engineeering, Beijing Institute of Technology, Beijing 100081, China; wangyq22@126.com (Y.W.); chenbit@126.com (S.C.); kevyu_bit@163.com (Z.Y.); jinshaohua@bit.edu.cn (S.J.); lilijie2003@bit.edu.cn (L.L.); 2 Institute of Chemical Materials, China Academy of Engineering Physics, Mianyang 621900, China; lixin.bit@163.com; 3 Research Institute, Gansu Yin’guang Chemical Industry Group Co., Ltd., Baiyin 730900, China; 364393124@163.com

**Keywords:** reduced sensitivity RDX, recrystallization, spheroidization, crystal morphology, shock sensitivity

## Abstract

The internal defects and shape of cyclotrimethylenetrinitramine (RDX) crystal are critical parameters for the preparation of reduced sensitivity RDX (RS-RDX). In the current study, RDX was re-crystallized and spheroidized to form the high-quality RDX that was further characterized by purity, apparent density, size distribution, specific surface area, impact sensitivity, and shock sensitivity. The effects of re-crystallization solvent on the growth morphology of RDX crystal were investigated by both theoretical simulation and experiment test, and consistent results were obtained. The high-quality RDX exhibited a high purity (≥99.90%), high apparent density (≥1.811 g/cm^3^), spherical shape, and relatively low impact sensitivity (6%). Its specific surface area was reduced more than 30%. Compared with conventional RDXs, the high-quality RDX reduced the shock sensitivities of PBXN-109 and PBXW-115 by more than 30%, indicating that it was a RS-RDX. The reduced sensitivity and good processability of the high-quality RDX would be significant in improving the performances of RDX-based PBXs.

## 1. Introduction

Cyclotrimethylenetrinitramine (RDX, C_3_H_6_N_6_O_6_), known as a high performance energetic material, has been widely utilized in the national defense industry. In recent years, many efforts have been made in the study of reduced sensitivity RDX (RS-RDX). Compared with the equivalent formulation of conventional RDX, that of RS-RDX can significantly reduce the shock sensitivity of cast-cured polymer bonded explosives (PBXs) [1,2].

At the crystal level, it has been reported that the internal crystal defects, mainly referred to voids and impurities, are the most important parameters determining the shock sensitivity of an explosive [3,4,5,6]. Re-crystallization is one of the most effective solutions to decrease such internal defects. Solvents and re-crystallization conditions are the critical factors affecting the crystal defects of RDX [7]. Recently, molecular dynamic simulation has been demonstrated as an important and reliable tool to investigate the influences of solvent on the crystal morphology of energetic materials including RDX and cyclotetramethylenetetranitramine (HMX) [8,9,10,11].

In addition to the processability, the shape of explosive particles may also be able to significantly affect the shock sensitivity of explosives by influencing the quality of the cast PBXs [6]. RDX can be spheroidized via erosion and partial dissolution under physical agitation. The corners and angles are preferentially dissolved by moderate intermolecular collision to generate spherical particles [12].

Up to now, to the best of our knowledge, there are only a few comprehensive studies on the preparation of RS-RDX. In the present work, we systematically studied the preparation of high-quality RDX containing spherical particles with minor internal defects by the combination of theoretical simulation and experiments. The interactions between solvent molecules and RDX crystal surfaces were investigated at the molecular level by molecular modeling, aiming to provide theoretical supports for RDX crystal morphology controlling. The growth morphologies of RDX crystals were predicted using the interfacial models between RDX faces and solvents constructed in the Growth Morphology Module of Materials Studio, which, along with the experimental results, were used to optimize the re-crystallization solvent of RDX. The re-crystallized RDX was further spheroidized to yield high-quality RDX. In addition to purity, apparent density, size distribution, specific surface area, and impact sensitivity, the obtained high-quality RDX was also characterized by shock sensitivity to confirm its reduced sensitivity. 

## 2. Results and Discussion

### 2.1. Validity of Force Field

The advanced COMPASS [13] force field was used to investigate the intermolecular interactions between RDX and solvent molecules. The validity of the COMPASS force field was tested by the accuracy of predicted static lattice energy (*E*_L_) of the crystal. *E*_L_ can be estimated with the experimental enthalpy of sublimation (−Δ*H*_sub_) by Equation (1) [14]

(1) $$E_{L}=-\Delta H_{\mathrm{sub}}-2\mathrm{RT}$$

where the last term represents the approximate correction to the difference between the gas-phase enthalpy, PV + 3RT (ideal gas), and the estimated vibrational contribution, 6RT. Due to the approximation embedded in Equation (1) and the experimental uncertainty, the error bar was estimated to be in the range of 3–4 kcal/mol. Clearly, the lattice energy given in Equation (1) corresponds to the idealized potential energy at zero temperature. Therefore, the reasonable validation can be conducted by comparing the lattice energy predicted by the energy minimization method against the experimental data. The experimental enthalpy of sublimation of RDX is 31.1 kcal/mol [15], and the predicted lattice energy of RDX is 28.6 kcal/mol, which gives an uncertainty of 8%, within the acceptable range of practical experiments. Therefore, it is proper and reliable to apply the COMPASS force field to the RDX crystal.

### 2.2. Simulated Growth Morphology of RDX in Vacuum

**Figure 1:**
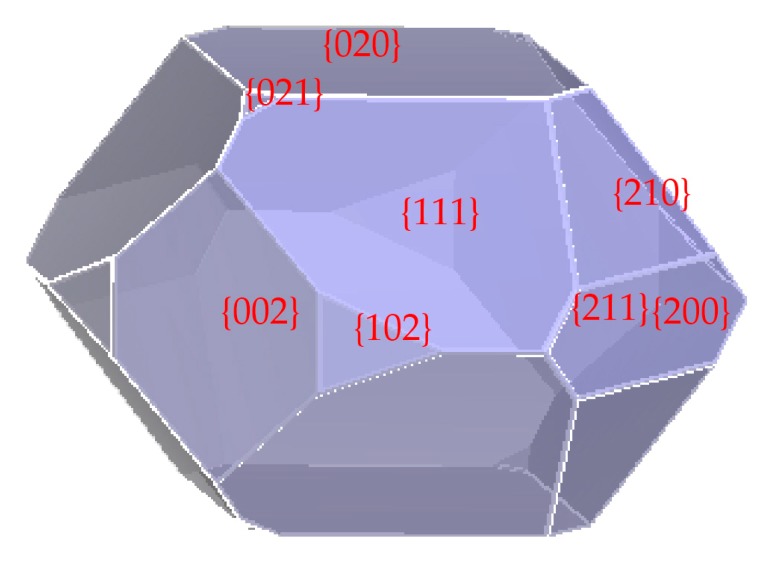
The morphology of RDX crystal grown in vacuum.

The morphology of RDX crystal grown in vacuum by the Attachment Energy (AE) theory is shown in Figure 1. Table 1 lists the characteristic data of the most probable crystal faces. It is clear that both attachment energy (*E*_att_) between RDX layers and area ratio vary significantly among these most probable crystal faces. {211} and {021} faces exhibited higher *E*_att_ and lower area ratios, while {111} and {020} faces showed lower *E*_att_ and higher area ratios. The length/diameter ratio (L/D) of the RDX crystal was calculated using Materials Studio (MS 6.0; Accelrys, San Diego, CA, USA) to be 1.66, indicating that the spherical degree of RDX crystal grown in vacuum was low.

### 2.3. Calculated Interaction Energy (E_int_) between RDX and Solvent

**Figure 2:**
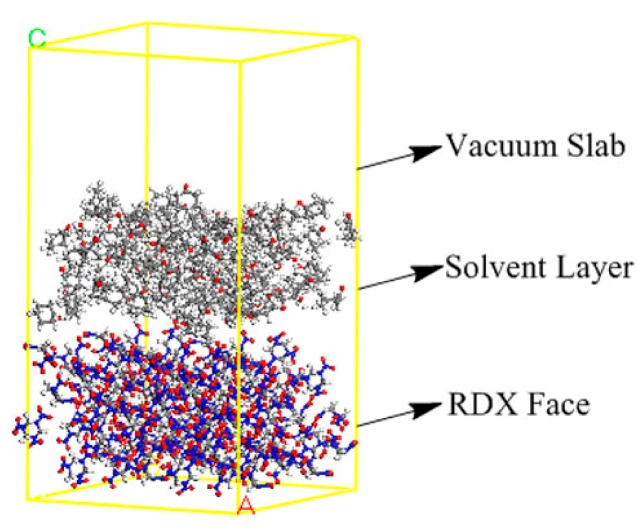
The typical interfacial model between a RDX face and solvent molecules.

The typical interfacial model between a RDX face and solvent molecules is shown in Figure 2. Table 2 lists the calculated interaction energy (*E*_int_) between each RDX face and solvent molecules. The $E_{\mathrm{att}}^{s}$ of RDX faces in the selected solvents were then calculated by Equation (3) using the corresponding *E*_att_ and *E*_int_ listed in Table 1 and Table 2 (seen Section 3.1). 

As shown in Table 3 for the parameters of each face of RDX, face {111} exhibited the highest area ratio, 50% more than those of other faces in all tested solvents except for GBL. In contrast, faces {200} and {002} disappeared. The $E_{\mathrm{att}}^{s}$ of face {111} is the lowest and those of faces {200} and {002} are the highest. According to the AE theory, a face with lower $E_{\mathrm{att}}^{s}$ possesses a high area ratio, and vice versa, the face with higher $E_{\mathrm{att}}^{s}$ has a lower area ratio or even disappears. In all, the $E_{\mathrm{att}}^{s}$ and area ratio of a crystal should be in a good consistence.

**Figure 3:**
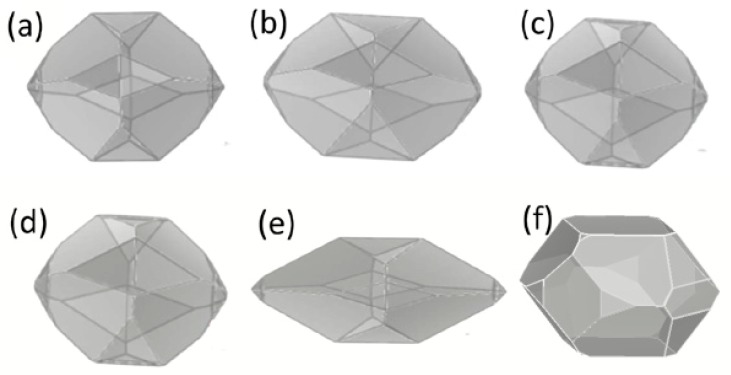
The growth morphology of RDX in solvents including acetone (**a**); cyclohexanone (**b**); γ-butyrrolacton (**c**); dimethyl sulfoxide (**d**); and *N*-methyl-2-pyrrolidone (**e**); vacuum (**f**) respectively.

The growth morphologies and length/diameter ratios (L/D) of RDX crystal in five selected solvents are shown in Figure 3 and Table 4, respectively. Compared with those grown in vacuum, both growth morphology and length/diameter ratio were slightly changed in all tested solvents; except for NMP where both growth morphology and length/diameter ratio of RDX underwent significant changes, resulting in a low spherical degree. The highest area ratio face {111} shown in Table 1 indicates it is the most important crystal face. A comparison between the attachment energies listed in Table 3 suggests that the $E_{\mathrm{att}}^{s}$ of {111} face with NMP is lowest not only among those of {111} faces with different solvents, but also among those of different faces with NMP. Consistent with the AE theory that the face with lower $E_{\mathrm{att}}^{s}$ possesses higher area ratio, the {111} face re-crystallized in NMP exhibited the highest area ratio with a value of over 85%. The exorbitant area ratio caused a significantly different growth morphology and length/diameter ratio of RDX in NMP and a lower spherical degree. 

Based on the discussion above, it can be concluded that acetone (AC), cyclohexanone (CH), γ-butyrrolactone (GBL), and dimethyl sulfoxide (DMSO) are more appropriate re-crystallization solvents for RDX than *N*-methylpyrrolidone (NMP).

### 2.4. Re-Crystallization of RDX in Selected Solvents

**Figure 4:**
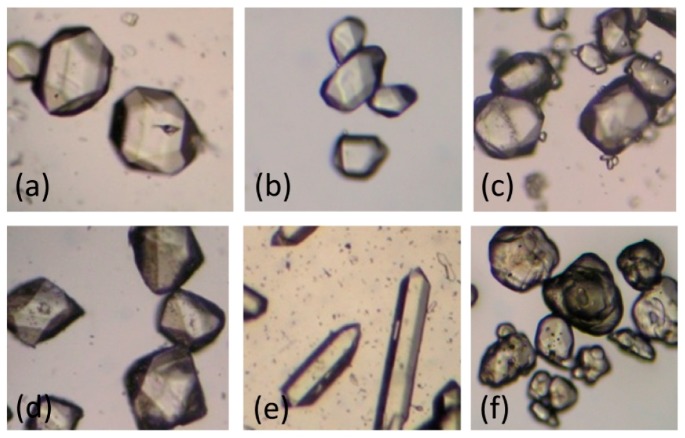
The photos of conventional RDX and RDX crystal from five solvents including acetone (**a**); cyclohexanone (**b**); γ-butyrolactone (**c**); dimethyl sulfoxide (**d**); and *N*-methyl-2-pyrrolidone (**e**) ; conventional RDX (**f**) respectively.

Figure 4 and Table 5 show the morphologies, purities, and apparent densities of conventional RDX and RDX recrystallized in the five selected solvents. All of the re-crystallized RDX exhibited more regular morphologies than conventional RDX with minor agglomerations (Figure 4). The RDX re-crystallized in AC, CH, DMSO, and GBL showed similar morphologies, while that re-crystallized in NMP displayed a different, rod-like crystalline morphology. These experimental results are in good agreement with the simulation results.

The purity and apparent density of the re-crystallized RDX are higher than those of conventional RDX (Table 5). The extremely high purity (≥99.90%) and apparent density (≥1.811 g/cm^3^) of the RDX crystal re-crystallized in AC and CH suggested its less internal defects [16], i.e., impurities and voids.

Based on these results, it can be concluded that AC and CH are the relatively better re-crystallization solvents of RDX.

### 2.5. Spheroidization of RDX

**Figure 5:**
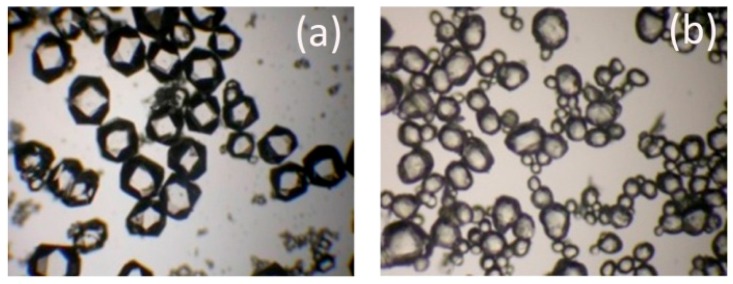
The microscopic photos of RDX samples after recrystallization (**a**); recrystallization and spheroidization (**b**).

The RDX re-crystallized in CH was further spheroidized in AC. Figure 5 shows the microscopic photos of RDX samples respectively treated by re-crystallization only, and re-crystallization followed by spheroidization. 

**Figure 6:**
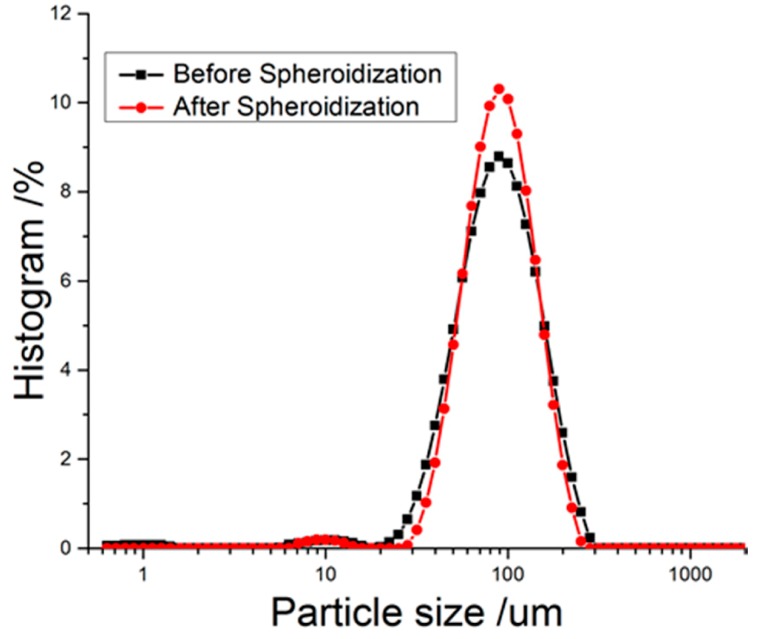
The size distribution of RDX before and after spheroidization.

Compared with the RDX re-crystallized in CH (Figure 5a), those re-crystallized in CH and further spheroidized in AC (Figure 5b) exhibited a higher spherical degree due to the dissolution-precipitation principle, by which the edges of the crystal were preferentially dissolved in solvent [17]. The percussion by solvent and friction between crystal particles under mechanical agitation deactivated the edges, which also contributed to the spheroidization of RDX. Figure 6 and Table 6 show the size distribution and specific surface area of the RDX after spheroidization treatment. It is clear that spheroidization significantly narrowed the crystal size distribution and was able to reduce the specific surface area by more than 30% on average.

### 2.6. Sensitivity of RDX

Compared with that of conventional RDX, the impact sensitivity of high-quality RDX descended from 30% to 6% (Table 7), possibly due to its less internal defects and higher spherical degree. In addition, its probability of hot spots arising under impact was also reduced.

PBXN-109 [18] and PBXW-115 [19] were selected as the vehicles to assess the relative shock sensitivities of the conventional RDX and high-quality RDX. The shock sensitivities of the PBXs were measured by the large scale gap test. Seen from Table 8, substituting the conventional RDX with the high-quality RDX reduced the number of cards from 99 to 69 for PBXN-109 and from 91 to 59 for PBXW-115. The evident reduction in shock sensitivity, 30% for PBXN-109 and 35% for PBXW-115, is consistent with the LSGT results reported by the Defense Science and Technology Organization (DSTO) that I-RDX was able to reduce shock sensitivity of PBXW-115 by 35% [19]. These results suggest that our high-quality RDX is a RS-RDX.

## 3. Materials and Methods

### 3.1. Computation

Molecular dynamics (MD) simulations were conducted in the Forcite module that was implemented in Materials Studio software 6.0. All MD simulations were carried out in the NVT ensemble (short for Canonical ensemble) at room temperature using the COMPASS force field. No additional symmetry constraints were used, except for the periodic boundary conditions imposed on simulation supercells. Initial velocities were chosen based on the Maxwell–Boltzmann profiles at given temperatures. Temperatures were selected by the stochastic collision method of Andersen. For potential energy calculations, the long-range Coulombic interactions were calculated by the standard Ewald method. VDW interactions (Lennard-Jones potential) were truncated at the cutoff of 0.95 nm, and the tail corrections to potential energy and pressure were included in the calculation.

The initial RDX structure used for the condensed phase simulation was adopted from the work of Choi et al. [20]. The eight molecules per unit cell RDX crystal was constructed with the lattice parameters *a* = 1.3444 nm, *b* = 1.1279 nm, *c* = 1.0221 nm, and α = β = γ = 90°. The Attachment Energy (AE) theory was used to predict the crystal morphology in vacuum with a list of the most probable crystal faces that appeared in the external morphology [21,22]. The RDX crystal was then cleaved along the predicted {hkl} face into periodic superstructures of 2 × 2 × 3 unit cell and a solvent layer containing 100 randomly distributed solvent molecules was constructed by the Amorphous Cell tool. A 50 Å thick vacuum slab was built above the solvent layer to eliminate the effect of additional free boundaries on the model. The interfacial model was optimized to achieve the equilibrium conformations for MD simulations (2 ns at time step 1 fs at 303 K with Andersen thermostat).

Based on the total energy of double layer construction (*E*_tot_), the RDX surface energy (*E*_surf_), solvent energy (*E*_solv_), and interaction energy between the RDX surface and solvent molecules (*E*_int_) can be calculated by Equation (2) [9,10].

*E*_int_ = *E*_tot_ − *E*_surf_ − *E*_sol_ (2)

The crystal morphology of RDX is significantly affected by solvent due to the interfacial interactions between RDX surface and solvent molecule. Therefore, the attachment energy ($E_{\mathrm{att}}^{s}$) in the selected solvent was calculated by Equation (3).

(3) $$E_{\mathrm{att}}^{s}=E_{\mathrm{att}}-E_{\mathrm{int}}$$

### 3.2. Experiment

#### 3.2.1. Materials

The conventional RDX, manufactured by the Woolwich process of hexamine or hexamethylenetetramine (HMT) with concentrated nitric acid, was supplied by Gansu Yin’guang Chemical Industry Group Co., Ltd. (Baiyin, China). Other reagents were all analytical grade.

#### 3.2.2. Re-Crystallization of RDX

Fifty grams of conventional RDX (D50 ≈ 100 μm) were added to a three-neck flask containing a desired solvent under agitation at 200–300 rpm and slowly heated to a temperature slightly below the boiling point of the solvent in a water bath until RDX was completely dissolved. The agitation speed was then reduced to 80–100 rpm. The ultrafine seed of RDX crystal (D50 ≈ 3 μm, 0.2 g) was added into the solution when a small amount of crystals were observed. The solution was cooled to ambient temperature at a cooling rate of 0.5 °C/min to re-crystallize the RDX. After the re-crystallization was completed, the RDX crystals were filtrated, washed, and dried.

#### 3.2.3. Spheroidization of RDX

The RDX re-crystallized in cyclohexanone (CH) was shperoidized using a saturated solution in AC under stirring at 200–300 r/min for 2–4 h until spherical shape particles were observed under microscope. The obtained high-quality RDX was filtrated, washed, and dried for further use.

#### 3.2.4. Preparation of PBXN-109 and PBXW-115

PBXN-109 [18] and PBXW-115 [23] were prepared to compare the properties of different RDX. TDI was selected as the curing agent. Plasticizer DOS, HTPB (including the bonding agent), TDI, Al, and RDX were sequentially added to a kneader, mixed for 20 min, vacuumed at 40 °C to exhaust the air, and cured at 60 °C in a PTFE mold for seven days. The obtained PBXN-109 contained 64% RDX, 20% Al, and 16% HTPB binder, and PBXW-115 consisted of 43% ammonium perchlorate (AP), 25% Al, 20% RDX, and 12% HTPB binder. Both formulations were prepared with conventional RDX and high-quality RDX for the comparison purpose. 

#### 3.2.5. Density Measurement

The apparent density of RDX was measured by the density gradient method [24]. Zinc bromide aqueous solutions with densities ranging from 1.780 g/cm^3^ to 1.815 g/cm^3^ in the gradient of 0.001 g/cm^3^ were prepared. RDX was dispersed in the mixture solutions and stirred for 2 h in a water bath at 25 ± 0.05 °C. The apparent density of RDX equals the density of the zinc bromide solution where RDX floats without any suspension or other force needed, except for the buoyancy in the liquid.

#### 3.2.6. Purity Measurement

The purity of RDX was measured using a Varian 5000 high performance liquid chromatograph (HPLC) equipped with a C18 chromatographic column. Methanol, acetonitrile, and water (28:12:60. *v*/*v*/*v*) were used as the mobile phase. The flow rate was set to 1.7 mL/min, and the pressure was 30 MPa. The inject volume was 5 μL. UV detection wavelength was 240 nm. 

#### 3.2.7. Size Distribution and Specific Surface Area Measurements

The size distribution of RDX was measured with a 1064 laser particle size analyzer (CILAS, Orléans, France). Its specific surface area was determined by the Brunauer, Emmet, and Teller (BET) method using an ASAP 2010M nitrogen adsorption apparatus (Micromeritics, Norcross, GA, USA).

#### 3.2.8. Sensitivity Test

The impact sensitivity was measured by the drop-hammer test using a 2.000 ± 0.002 kg hammer that was dropped from a height of 25 cm. Fifty tests were conducted on 30 ± 1 mg samples and the results were reported as the probability of explosion (%).

The shock sensitivity of PBXs containing either high-quality RDX or conventional RDX was determined by the large scale gap test according to the 605.1 method listed in GJB 772A-97 of China. The gap material (PMMA, diameter: 50 mm, thickness: 0.5 mm) was placed between the donor (melt-cast explosives consisting of 50 wt % PETN and 50 wt % TNT, diameter: 50 mm, height: 50 mm) and the acceptor (RDX-based PBX, diameter: 36 mm, height: 140 mm). The number of cards required for a 50% chance to detonate the tested explosive with the output from the donor charge was determined.

## 4. Conclusions

The preparation of high-quality RDX was systematically studied by the combination of theoretical simulation and experimental tests. The MD simulation and experiment tests gave consistent results on the effects of re-crystallization of solvent on the growth morphology of RDX crystal. The RDX crystals re-crystallized in acetone and cyclohexanone exhibited a regular shape, high purity (≥99.90%), and high apparent density (≥1.811 g/cm^3^), indicating that acetone and cyclohexanone were optimum re-crystallization solvents. The re-crystallized RDX was further spheroidized to produce high-quality RDX with a higher spherical degree, at least 30% smaller specific surface area, and lower impact sensitivity (6%) than conventional RDX. Compared with the conventional RDX, the high-quality RDX was able to reduce the shock sensitivities of PBXN-109 and PBXW-115 more than 30%, indicating that it was a RS-RDX. In addition, the high-quality RDX displayed a good processability, which, along with its reduced sensitivity property, is of great significance in improving the performances of RDX based PBXs.

## Figures and Tables

**Table 1:** The face characteristic data of RDX growing in vacuum

{hkl}	N ^1^	*E*_att_ (kcal·mol^−1^)	*S* ^2^ (nm^2^)	Area Ratio ^3^ (%)
{111}	8	−14.3	1103.5	52.5
{020}	2	−11.9	420.8	20.0
{210}	4	−16.8	210.1	10.0
{002}	2	−14.4	200.4	9.5
{200}	2	−17.8	110.4	5.2
{102}	4	−16.0	48.4	2.3
{021}	4	−15.5	6.2	0.3
{211}	8	−17.5	2.6	0.1

Note: ^1^ the number of certain face {hkl}; ^2^ the area of certain face {hkl}; ^3^ the area of certain face {hkl} divided by whole face area of the crystal.

**Table 2:** The calculated *E*_int_ between each RDX faces and solvent molecules.

{hkl}	*E*_int_ (kcal·mol^−1^)				
	AC	CH	DMSO	GBL	NMP
{111}	−7.4	−8.0	−7.0	−8.4	−9.2
{020}	−4.0	−3.6	−3.7	−4.2	−4.6
{210}	−8.0	−7.9	−6.9	−8.7	−7.9
{002}	−3.1	−3.7	−3.2	−4.0	−4.0
{200}	−4.5	−4.5	−4.1	−5.1	−4.6
{102}	−8.7	−8.8	−8.9	−10.3	−9.7
{021}	−9.2	−9.2	−8.0	−9.3	−9.8
{211}	−9.3	−9.0	−8.6	−10.7	−10.1

**Table 3:** The $E_{\mathrm{att}}^{s}$ and area ratio of each face of RDX in selected solvents.

{hkl}		{111}	{020}	{210}	{002}	{200}	{102}	{021}	{211}
**(kcal·mol^−1^)**	**Vacuum**	−14.3	−11.9	−16.8	−14.4	−17.8	−16	−15.5	−17.5
	**AC**	−6.9	−7.9	−8.8	−11.3	−13.3	−7.2	−6.3	−8.2
	**CH**	−6.4	−8.4	−8.9	−10.7	−13.3	−7.2	−6.3	−8.6
	**DMSO**	−7.3	−8.3	−9.9	−11.2	−13.7	−7.1	−7.5	−9
	**GBL**	−6	−7.7	−8.1	−10.4	−12.6	−5.6	−6.2	−6.8
	**NMP**	−5.2	−7.3	−8.9	−10.4	−13.2	−6.3	−5.7	−7.4

**Table 4:** Calculated length/diameter ratio of RDX crystal.

Solvent	AC	CH	DMSO	GBL	NMP	Vacuum
L/D	1.62	1.67	1.62	1.61	1.93	1.66

**Table 5:** The purity and apparent density of RDX.

Parameters	Recrystallized RDX					Conventional RDX
	AC	CH	GBL	DMSO	NMP	
**Purity (%)**	99.90	99.92	99.71	99.76	99.85	99.27
**ρ (g·cm^−3^)**	1.813	1.811	1.806	1.803	1.809	1.796

**Table 6:** The specific surface area of RDX before and after spheroidization

Experiment Number	Specific Surface Area (m^2^·g^−1^)	
	Before	After
1	0.06739	0.04585
2	0.07658	0.04637
3	0.07269	0.04602
Average	0.07222	0.04608

**Table 7:** The impact sensitivity of RDX.

RDX	Impact Sensitivity (%)
Conventional RDX	30
High-quality RDX	6

**Table 8:** The shock sensitivity of RDX-based cast-cured PBXs.

Cast Cured PBX	RDX Source	Number of Cards
PBXN-109-1	Conventional RDX	99
PBXN-109-2	High-quality RDX	69
PBXW-115-1	Conventional RDX	91
PBXW-115-2	High-quality RDX	59

## References

[1] Johansen Ø.H., Kristiansen J.D., Gjersøe R., Berg A., Halvorsen T., Smith K. (2008). RDX and HMX with Reduced Sensitivity Towards Shock Initiation—RS-RDX and RS-HMX. Propellants Explos. Pyrotech..

[2] Spyckerelle C., Eck G., Berg P.S., Amnéus A. (2008). Reduced Sensitivity RDX Obtained From Bachmann RDX. Propellants Explos. Pyrotech..

[3] Doherty R.M., Watt D.S. (2008). Relationship between RDX Properties and Sensitivity. Propellants Explos. Pyrotech..

[4] Borne L., Patedoye J., Spyckerelle C. (1999). Quantitative Characterization of Internal Defects in RDX Crystals. Propellants Explos. Pyrotech..

[5] Oxley J., Smith J., Buco R., Huang J. (2007). A Study of Reduced-Sensitivity RDX. J. Energ. Mater..

[6] Van der Heijden A.E.D.M., Bouma R.H.B., van der Steen A.C. (2004). Physicochemical Parameters of Nitramines Influencing Shock Sensitivity. Propellants Explos. Pyrotech..

[7] Kim J., Kim J., Kim H., Koo K. (2009). Characterization of Liquid Inclusion of RDX Crystals with a Cooling Crystallization. Cryst. Growth Des..

[8] Chen G., Xia M., Lei W., Wang F., Gong X. (2013). A study of the solvent effect on the morphology of RDX crystal by molecular modeling method. J. Mol. Model..

[9] Chen H., Li L., Jin S., Chen S., Jiao Q. (2012). Effects of Additives on ε-HNIW Crystal Morphology and Impact Sensitivity. Propellants Explos. Pyrotech..

[10] Duan X., Wei C., Liu Y., Pei C. (2010). A molecular dynamics simulation of solvent effects on the crystal morphology of HMX. J. Hazard. Mater..

[11] Wang D., Chen S., Li Y., Yang J., Wei T., Jin S. (2013). An Investigation into the Effects of Additives on Crystal Characteristics and Impact Sensitivity of RDX. J. Energ. Mater..

[12] Lavertu R.R.S., Godbout A.C. (1977). Process for Spheroidization of RDX Crystals. U.S. Patent.

[13] Sun H. (1998). COMPASS: An ab Initio Force-Field Optimized for Condensed-Phase Applicationss—Overview with Details on Alkane and Benzene Compounds. J. Phys. Chem. B.

[14] Pertsin A.J. (1987). The Atom-Atom Potential Method.

[15] Zhu W., Xiao J., Zhu W., Xiao H. (2009). Molecular dynamics simulations of RDX and RDX-based plastic-bonded explosives. J. Hazard. Mater..

[16] Borne L., Beaucamp A. Effects of Explosive Crystal Internal Defects on Projectile Impact Initiation. Proceedings of the 11th International Detonation Symposium, Snowmass Conference Center.

[17] Nguyen A.M., Nordborg A., Shchukarev A., Irgum K. (2009). Thermally induced dissolution/precipitation—A simple approach for the preparation of macroporous monoliths from linear aliphatic polyamides. J. Sep. Sci..

[18] Lochert I.J., Dexter R.M., Hamshere B.L. (2002). Evaluation of Australian RDX in PBXN-109.

[19] Lochert I.J., Franson M.D., Hamshere B.L. (2003). Reduced Sensitivity RDX Part I: Literature Review and DSTO Evaluation.

[20] Choi C.S., Prince E. (1972). The Crystal Structure of Cyelotrimethylene-trinitramine. Acta Cryst..

[21] Hartman P. (1980). The Attachment energy as a habit controlling factor III. application to corundum P. hartman. J. Cryst. Growth.

[22] Hartman P. (1980). The attachment energy as ahabit controlling factor II. Application to anthracene, tin tetraiodide and orthorhombic sulphur P. Hartman. J. Cryst. Growth.

[23] Lu J.P., Kennedy D.L. (2003). Modelling of PBXW-115 Using Kinetic Cheetah and the Dyna Codes.

[24] Hoffman D.M. (2003). Voids and Density Distributions in 2,4,6,8,10,12-Hexanitro-2,4,6,8,10,12-Hexaazaisowurtzitane (CL-20) Prepared Under Various Conditions. Propellants Explos. Pyrotech..

